# Supplementary material for: Offset nail fixation for intertrochanteric fractures improves reduction and lag screw position
Source: PLoS One. 2022 Nov 16;17(11):e0276903. doi: 10.1371/journal.pone.0276903 (PMC9668128; doi:10.1371/journal.pone.0276903)
Supplement: Attachment — Submitted filename: Response to reviewers.docx [file pone.0276903.s002.docx]

Response to Editor and Reviewers

**PONE-D-22-22038**

**Offset nail fixation for intertrochanteric fractures improves reduction and lag screw position**

Dear Editor and Reviewers

We thank the Editor and Reviewers for thoughtful evaluation of our manuscript. We have given careful attention to the suggestions and addressed their comments. Point-by-point responses to specific comments are provided below. The editor’s and reviewers’ comments are in bold, italic and underlined. The changes made in the manuscript were highlighted.

To Editor

***PLOS only allows data to be available upon request if there are legal or ethical restrictions on sharing data publicly.***

→ We uploaded our raw data as a Supporting Information file, accordingly.

To Reviewer #1:

General

1) ***Is there any published data detected a racial variation in the relation between the neck and shaft of the femur? Are the results obtained from the current study applicable for non– Japanese patients? The authors should discuss this point in the “Discussion”.***

→ Thank you for the valuable comments. In fact, only a few studies investigated the offset of the femoral neck or its racial differences. Anastopoulos reported the 3.5 ± 1.5 mm offset in Greek patients (Reference #20). Bonneau et al. reported the 4.9 ± 2.6 mm offset in 50 French and Swiss cases (Reference #21). Cornelissen reported the 6.1 ± 1.7 mm offset in 100 African cases (Reference #22). These studies show that femoral neck offsets are comparable among various racial groups. We discussed this point in the Discussion section (page 10 of the revised manuscript).

2) ***As the authors of the current manuscript are the developer of the (Best Fit Nail® [BFN]) (Line 74), I wander why they did not plan the study to be a randomized controlled study (RCT) instead of the current retrospective study (with its limitations).***

→ Thank you for your comment. In this paper, we tried to evaluate the usefulness of BFNs in maintaining the reduction of fragments and ensuring proper lag screw positioning, compared to that with conventional non-offset nails (PFNA) retrospectively. We agree with the reviewer that it is important to confirm the results in this study in an RCT setting in the future.

3) ***Data presented in the manuscript are incomplete and might be deceiving. The results of the manuscript prove that BFN exhibited significantly post-operative smaller displacement distance, better reduction status, and higher frequency of no deviation, with central lag screw positioning, compared to those using non-offset PFNA. What about the differences at the end of follow up? What about the effects of these differences on the clinical results and patient reported outcomes?***

→ Thank you for the valuable comments. As the reviewer pointed, it is important to elucidate the clinical significance between BFN and non-off set nails. So far, we have only preliminary results that patients treated with BFN return to daily activities somewhat faster, which needs to be confirmed with more cases in the future.

4) ***Line 39-40: “The median displacement distance [interquartile range] was significantly smaller in the BFN group (0 [0, 0] mm) and in the PFNA group (5.2 [3.6, 7.1] mm)” should be corrected to “The median displacement distance [interquartile range] was significantly smaller in the BFN group (0 [0, 0] mm) than in the PFNA group (5.2 [3.6, 7.1] mm)”***

→ Corrected, accordingly.

5) ***Line 185: “To overcome these difficulties, we developed IHNs with a selective anterior…” This is a continuation of the previous paragraph and should not be a new one.***

→ We removed the phrase, accordingly.

To Reviewer #2

1) ***The authors mentioned that the Study was conducted retrospectively. Were the cases consequent? How did they avoid selection bias?***

→ Consecutive patients who underwent surgery for intertrochanteric fracture using BFNs or using non-offset PFNA nails were included in this study (page 5), and we did not exclude the bias in advance. However, when the background data were compared, there were no significant differences in age, sex, height, weight, or AO classification between the BFN and PNFA groups as a result, which may be due to the relatively consistent indications for surgery.

2) ***The authors conducted CT-Examination for all the patients included in the study. I wonder what was the indication to perform a CT-scan for the patients which is not a routine post operative examination for this type of fractures. This point raises an important ethical consideration: what was the justification to expose the patients to the hazards of CT radiation over the routine postoperative X ray. What consequences did the CT had on the Patient Treatment?***

→ Thank you for raising an important issue. As the reviewer mentioned, it is important to avoid radiation exposure to the patients. However, CT examination is very common and reimbursed in Japan not only in orthopedic practice but also other medical departments partly because of the high prevalence of CT equipment. In addition, we consider accurate evaluation of the displacement of fractures by CT is very important for planning postoperative rehabilitation. We are trying to minimize the radiation exposure to the patients as much as possible, and routine post-operative blood tests revealed there were no cases of radiation-related complications.

3) ***It is well known to all orthopedic and trauma surgeon that the reduction of the intertrochanteric fractures on the traction table is performed before starting the implantation of the nail. The nail fixes only the achieved reduction. How could the authors Proof that the better reduction is related to the nail itself (not the surgical technique) specially when the 2 nails are performed in 2 different clinics by different surgeons.***

→ As the reviewer pointed out, it is possible that the better reduction was caused by the surgical technique and not by the difference of nails. However, all the surgeries were performed by surgeons specializing in fracture surgery, and there was a clear difference in the post-operative reduction status between BFN group and PFNA group. Therefore, we consider that this difference is due to the different design of the nails.

4) ***The authors mentioned that there were no adverse effect related to the performed surgeries. this is a broad word mentioned in the results without mentioning how did they look for this in the methodology. Which type of adverse effects were Evaluated (Clinical/ Radiological) ? Intraoperative or Post operative? How was the evaluation of the adverse effects was conducted? how long was the follow up to mention this fining in the outcome?***

→ Thank you for your comments. Clinical (and radiological) adverse events reported by surgeons, such as postoperative infection, venous thromboembolism, and perioperative fractures were counted as adverse events in this study. This is explained in the revised manuscript (page 9 of the revised manuscript).

5) ***The article is written in a clear and simple English language. Yet there are some spelling, grammatical and punctuation mistakes:***

***­ Line 34: Antirotatin should be antirotation***

***­ Line 152: one case not 1 cases.***

***­ I recommend the article and the punctuation to be revised by an english language specialist.***

→ We corrected the typos, accordingly. The article was edited by an English-speaking specialist.

To Reviewer #3:

***I think this new design should be used in a wider scale of patients before popularization of the design and technique, however many commercial issues will affect the progress of popularization of the implants, good luck.***

→Thank you for the comments. We also recognize that many commercial issues will affect the progress of popularization of the implants as the reviewer mentioned.

**Other changes**

1) We made a mistake in the number of patients with 130 and 125 degrees of BFN nails, and corrected (page 9 and **Table 1**).
2) The mean age of patients in the PFNA group and the number of patients in each of the AO classifications in the BFN group were incorrect and were corrected (**Table 1**). The data were statistically re-analyzed and the p-values remained unchanged.

We are sorry for the mistakes.
