# Supplementary material for: Offset nail fixation for intertrochanteric fractures improves reduction and lag screw position
Source: PLoS One. 2022 Nov 16;17(11):e0276903. doi: 10.1371/journal.pone.0276903 (PMC9668128; doi:10.1371/journal.pone.0276903)
Supplement: Attachment — Submitted filename: Response to Editor and Reviewers.docx [file pone.0276903.s003.docx]

Response to Editor and Reviewers

**PONE-D-22-22038R1**

**Offset nail fixation for intertrochanteric fractures improves reduction and lag screw position**

Dear Editor and Reviewers

We thank the Editor and Reviewers for thoughtful evaluation of our manuscript. According to the Editor and Reviewers’ comments, we revised the manuscript. Point-by-point responses to specific comments are provided below. The editor’s and reviewers’ comments are in bold, italic and underlined. The changes made in the manuscript were highlighted.

To Editor

***I personally go through your manuscript. The topic is interesting but the manuscript is not written in intelligible fashion and I will suggest you to please re-write your manuscript in scientific language. English language can be further polished.***

→According to the comment, the revised manuscript was edited by an English-speaking scientist. I have not highlighted the text if it is only a wording or grammatical modification.

To Reviewer 2

1) ***The Authors answered the questions and commented of the reviewers but did not add their ideas in the manuscript (with few exceptions). For example, they did not add follow up Period, instead reported some few possible complication which was excluded clinically by the surgeons in the results section. They did not add the weak points mentioned by the authors in the discussion section. They commented on the hazards of radiation in their reply but did mention that in their manuscript. I recommend the authors to add their replay on the reviewers in the discussion section.***

→I apologize that I did not revise the manuscript satisfactorily. According to the reviewer’s comments, we added the following sentences in Results and Discussion sections.

“No clinical or radiographical adverse events related to surgery, such as postoperative infection, venous thromboembolism, and perioperative fractures, were observed in either group during the minimum follow-up period of 3 months.” (page 11)

“Fifth, it is possible that the better reduction in the BFN group was caused by the surgical technique and not by the difference in the nails used. However, all of the surgeries were performed by surgeons specializing in fracture surgery, and there was a clear difference in the postoperative reduction status between the BFN group and the PFNA group. Thus, we consider that the difference arose from the different designs of the nails.” (page 15-16)

“Although it is important to avoid unnecessary exposure of patients to radiation, CT examination is very common and reimbursed in Japan for not only orthopaedic departments but also other medical departments, partly due to the high prevalence of CT equipment in hospitals. Moreover, we consider that accurate evaluation of the fracture displacement by CT is very important for postoperative rehabilitation planning. We tried to minimize the radiation exposure to our patients as much as possible, and routine postoperative blood tests revealed that there were no cases of radiation-related complications.” (page 16)

2) ***Additionally I recommend the authors to omit the clinical follow up completely and emphasis in the aim of the study on that they were looking only on the CT finding postoperatively. this may make the article more plausible.***

→According to the reviewer’s comments, we removed the following sentences “We have preliminary results suggesting that the reduction of fragments and lag screw positioning were similar between PFNA and other non-offset nail cases, which should be further analyzed. (page 15)”
